# Evaluation of antioxidant and neuroprotective activities of *Cassia fistula* (L.) using the *Caenorhabditis elegans* model

**DOI:** 10.7717/peerj.5159

**Published:** 2018-07-13

**Authors:** Sara Thabit, Heba Handoussa, Mariana Roxo, Nesrine S. El Sayed, Bruna Cestari de Azevedo, Michael Wink

**Affiliations:** 1 Department of Pharmaceutical Biology, Faculty of Pharmacy and Biotechnology, German University in Cairo, Cairo, Egypt; 2 Department of Biology, Institute of Pharmacy and Molecular Biotechnology, Heidelberg University, Heidelberg, Germany; 3 Department of Pharmacology and Toxicology, Faculty of Pharmacy, Cairo University, Cairo, Egypt; 4 Departmento de Biotecnologia em Plantas Medicinais, Universidade de Ribeirão Preto, Ribeirão Preto, Brazil

**Keywords:** *Cassia fistula*, *C. elegans*, Antioxidant, Neuroprotection, Alzheimer, Huntington, HPLC/PDA/ESI-MS

## Abstract

**Background:**

*Cassia fistula* (L.) (Fabaceae) is a medicinal plant from tropical Asia. It is known for its marked antioxidant activity, which is attributed to its high phenolic content. The present study aims at testing both the antioxidant and neuroprotective effects of a hydroalcoholic extract from the aerial parts of *Cassia fistula* using the *Caenorhabditis elegans* model, which is widely used in this context.

**Methods:**

Chemical profiling of secondary metabolites that seem to be responsible for both antioxidant and neuroprotective capacities was carried out by HPLC/PDA/ESI-MS^n^. Antioxidant activity was tested *in vitro* by CUPRAC and DPPH assays. *In vivo* antioxidant and neuroprotective activities were investigated using the *C. elegans* model.

**Results:**

The *Cassia* extract improved the survival rate of the nematodes and protected them against oxidative stress. In addition, a decrease in the accumulation of reactive oxygen species (ROS) was observed. The important role of DAF-16/FOXO pathway was confirmed through an increased nuclear localization of the DAF-16 transcription factor, increased expression of SOD-3 stress response gene and decreased expression of HSP-16.2. Furthermore, the putative involvement of SKN-1/NRF2 pathway was demonstrated by a decrease in GST-4 levels. A neuroprotective activity of the *Cassia* extract was shown by a decline in polyglutamine (polyQ40) aggregate formation and a delay in paralysis caused by amyloid beta (Aβ_1–42_) accumulation.

**Discussion:**

The *Cassia* extract exhibits substantial antioxidant and neuroprotective activities *in vivo*, which might provide a rich and novel source of natural antioxidants and neuroprotective compounds to be further studied for the use in various food and cosmetic industrial fields.

## Introduction

Reactive oxygen species (ROS) are naturally generated in the body as they are necessary for several biological processes. However, high levels of ROS can lead to oxidative stress and damage of several macromolecules (Mittal et al., 2014). The loss of ROS homeostasis can induce mutations and therefore, ROS are considered as a major risk factor for highly prevalent age-related diseases such as cancer, cardiovascular diseases and neurodegenerative diseases, like Alzheimer’s, Parkinson’s and Huntington’s diseases (Lin & Beal, 2006; Forstermann, 2008; Reuter et al., 2010).

Antioxidant enzymes, like superoxide dismutase (SOD) and catalase (CAT), play an important role in defending the body against harmful effects of high ROS levels (Mates, Perez-Gomez & De Castro, 1999).

The ability of dietary antioxidants, especially polyphenols, to counteract ROS accumulation by scavenging free radicals and by modulating the expression of stress response related genes has been widely reported. Epigallocatechin gallate (EGCG), resveratrol and curcumin are examples of polyphenols that are well-known for their potent antioxidant activities (Abbas & Wink, 2009; Abbas & Wink, 2010; Alex et al., 2010).

Different *Cassia* species are characterized by being rich in phenolic constituents that correlate with their strong antioxidant activities. For example, a methanol extract of *Cassia abbreviata* roots demonstrated significant antioxidant and hepatoprotective activities. These activities were correlated to the high phenolic content of the plant, particularly catechins and proanthocyanidins (Sobeh et al., 2018). Previous studies on *Cassia alata* chemical composition have also shown a wide range of phenolic compounds including kaempferol, rhein, chrysophanol, aloe-emodin and physcion (Fernand et al., 2008). It has demonstrated considerable free radical scavenging effects (Sujatha & Asokan, 2017).

*Cassia fistula* (Fabaceae) is a medium sized tree, which originates from tropical Asia but is presently cultivated in many tropical areas of the Old and New World. In the traditional medicine it is used to treat liver problems, skin diseases and diabetes, in addition to being widely used as a laxative to treat obstipation. Moreover, antipyretic, analgesic, anti-inflammatory and hypoglycemic effects have been reported (Patel et al., 1965; Markouk et al., 2000; Bahorun, Neergheen & Aruoma, 2005).

Previous phytochemical analysis of *C. fistula* extracts revealed the presence of many phenolic compounds like flavonoids, anthraquinones and procyanidins, in addition to coumarins, alkaloids and several other glycosides (Morimoto et al., 1988; Fouad et al., 2018). These secondary metabolites are well-known for many biological effects including antioxidant, anti-inflammatory and anticarcinogenic activities (Middleton, Kandaswami & Theoharides, 2000). Sennosides A & B and free rhein were previously identified in the leaves of the plant while kaempferol and fistulin (bianthraquinone glycoside) were found in the flowers (Khare, 2008). Additionally, rhamnetin 3-*O*-gentiobioside was isolated from the roots (Vaishnav & Gupta, 1996) and the isoflavone biochanin A was isolated from the fruits (Sartorelli et al., 2009).

*C. fistula* extracts from different plant parts have demonstrated potent antioxidant activities. The aqueous flower extract was able to normalize the levels of some antioxidant enzymes like SOD, CAT and glutathione reductase in diabetic rats (Manonmani et al., 2005). A methanol fruit extract was able to inhibit 5-lipoxygenase resulting in inhibition of leukotriene B4 biosynthesis (KC & Muller, 1998). Furthermore, both the aqueous and methanol extracts of the bark previously showed significant radical scavenging effects and were able to inhibit lipid peroxidation caused by CCl_4_ and FeSO_4_ in rats (Ilavarasan, Malika & Venkataraman, 2006).

Moreover, the neuroprotective potential of a methanol extract of *C. fistula* roots was demonstrated by its ability to inhibit acetylcholinesterase (AChE), which is considered a promising target for the treatment of neurodegenerative disorders like Alzheimer’s disease (Rajasree, Singh & Sankar, 2012). Several studies demonstrate the strong correlation between antioxidant capacity and neuroprotective activity (Crotty, Ascherio & Schwarzschild, 2017; Velusamy et al., 2017; Bonfili et al., 2018). The previously demonstrated antioxidant capacity of *C. fistula* and its poorly understood neuroprotective potential makes it a good candidate for further investigations. According to a previous study, the antioxidant power and the total phenolic content of *C. fistula* is higher in stem barks, followed by leaves, flowers and pulps (Siddhuraju, Mohan & Becker, 2002). Based on that, leaves and fine stems of *C. fistula* were selected for the present study.

*Caenorhabditis elegans* is a nematode, which has been widely used as a model organism in the field of antioxidant and neuroprotection research because of its small size, easy way of culturing using *E. coli* as food source, rapid reproduction and short life cycle (Epstein & Shakes, 1995). Furthermore, the worm is transparent and it is easy to induce mutation in its genome. These two features allow the use of *in vivo* fluorescence markers, as Green fluorescent protein (GFP), to track important enzymes and cellular pathways like those involved in aging and oxidative stress (Kaletta & Hengartner, 2006). It is considered a good model for assaying neurodegenerative diseases due to the simplicity of its nervous system with only 302 neurons in adult worms (Wu et al., 2006).

This study aimed at testing the *in vivo* antioxidant and neuroprotective activities of a hydroalcoholic extract of *Cassia fistula* (CF) leaves and fine stems, using *C. elegans* as a model organism.

Initially, the extract was chemically characterized by HPLC/PDA/ESI-MS^n^ and its *in vitro* antioxidant effect was tested using CUPRAC and DPPH standard assays.

The ability of the extract to protect the nematodes from acute oxidative stress and to reduce intracellular ROS was studied. Furthermore, the involvement of DAF-16/FOXO transcription factor was tested together with the expression of heat shock proteins (HSP-16.2) and stress response genes, like superoxide dismutase-3 (SOD-3) and glutathione S-transferase-4 (GST-4).

The neuroprotective activity of CF was evaluated by testing its influence on the formation of polyQ40 aggregates, which are involved in Huntington’s disease and other proteinopathies (Williams & Paulson, 2008). Paralysis assays were also performed to evaluate whether CF is able to protect the worms against paralysis induced by Aβ plaque formation, a well characterized hallmark of Alzheimer’s disease (Liu et al., 2015).

## Materials and Methods

### Plant material and extraction

*C. fistula* leaves and fine stems were obtained during the spring (March and April) 2016 from Orman Botanical Garden, Giza, Egypt. Authenticity was confirmed by Professor Mohamed El Gebaly, Professor of Taxonomy at the National Institute of Research, Egypt.

Freshly harvested leaves and stems were homogenized and extracted using 70% v/v (methanol/water). The extract was then filtered through cotton wool followed by passing on charcoal to get rid of chlorophyll, then vacuum concentration at 37 °C using rotary evaporator was done (Nair, 1995). The concentrated filtrate was finally freeze-dried and the final product was stored at −20 °C for future utilization.

### Chemicals

Purified EGCG from green tea (purity ≥ 95%), 2,7-dichlorofluorescein diacetate (DCF-DA), 2,2-diphenyl-1-picrylhydrazyl (DPPH^•^), butylated hydroxyl anisole (BHA), bis (neocuproine) copper (II) chelate (CUPRAC reagent), vitamin C (Vit C) and juglone (5-hydroxy-1,4-naphthalenedione) were purchased from Sigma-Aldrich, Germany. Sodium azide was bought from AppliChem GmbH, Darmstadt, Germany; Folin-Ciocalteu reagent from Merck, Darmstadt, Germany.

### HPLC/PDA/ESI-MS^n^ qualitative chemical profiling

LC-MS/MS analysis was done on a Finnigan LCQ-Duo ion trap mass spectrometer with an ESI source (Thermo Quest, Austin, TX, USA) coupled to a Finnigan Surveyor HPLC system (MS pump plus, autosampler, and PDA detector plus) with an EC 150/3 NUCLEODUR 100-3 C18ec column (Macherey-Nagel, Düren, Germany).

A gradient of water and acetonitrile (ACN), without acid, was applied from 5% to 60% ACN in 45 min at 30 °C. Flow rate was 0.5 ml/min. Injection volume was about 20 µl. MS was operated in the negative mode with a capillary voltage of −10 V, source temperature of 220 °C, and high purity nitrogen as a sheath and auxiliary gas at a flow rate of 80 and 40 (arbitrary units), respectively. Ions were detected in a mass range of 50–2,000 m/z. Collision energy of 35% was used in MS/MS for fragmentation. Data acquisitions and analyses were done using Xcalibur™ 2.0.7 software (Thermo Fisher Scientific, Waltham, MA, USA).

### Folin-Ciocalteu assay for quantification of total phenolic content

The assay was performed in a 96-well microplate as described by (Zhang et al., 2006). Briefly, 100 µl of Folin-Ciocalteu reagent were added to 20 µl of the extract. The microplate was kept afterwards for 5 min at room temperature then 80 µl of sodium carbonate solution (7.5%) were added. The reaction was allowed to occur for 1 h in the dark at room temperature and then absorbance was recorded using microplate reader at 750 nm. Gallic acid was used as a standard to establish a calibration curve. The assay was repeated three times. Phenolic content is presented as µg of gallic acid equivalents (GAE)/mg of CF extract.

### CUPRAC method to assay total antioxidant capacity

The assay was carried out according to the method described by Apak et al. (2004). In a test tube, BHA standard or CF, 0.3 ml, were added to 0.8 ml purified water to reach a volume of 1.1 ml. To the previous mixture, 1 ml (10 mM) copper chloride, 1 ml (7.5 mM) neocuproine alcoholic solution and 1 ml of (1 M) ammonium acetate buffer were added to reach a final volume of 4.1 ml. Test tubes were closed and kept for 30 min, and then absorbance was recorded at 450 nm against a reagent blank using JASCO V-630 double beam spectrophotometer equipped with quartz cuvettes.

The assay was repeated three times. A calibration curve was drawn for BHA standard using a series of different concentrations (50, 100, 200 and 400 µg/ml) where absorbance was plotted versus concentration to get the 50% effective concentration of the standard antioxidant used, EC_50_, in µg/ml. Another calibration curve was plotted for CF using the same series of concentrations (50, 100, 200 and 400 µg/ml). EC_50_ was obtained for CF from the curve using the equation *y* = *ax* + *b* where y is substituted by 50% as follows:

\begin{eqnarray*}{\mathrm{EC}}_{50}= \frac{0.5-\mathrm{b}}{\mathrm{a}} \end{eqnarray*}

### DPPH assay

To assay the power of CF to scavenge radicals, DPPH free radical assay was used according to Blois method (Blois, 1958). The assay was done in a 96-well microplate. 100 µl of 200 µM DPPH were added to 100 µl of different concentrations of CF, Vit C or EGCG. Afterwards, incubation was done at room temperature in the dark for 30 min and absorbance was recorded at 517 nm using a microplate reader. The assay was repeated three times. The ability of CF and standard antioxidants to scavenge DPPH radicals was calculated as follows:

\begin{eqnarray*}\text{DPPH scavenging activity (%)}= \frac{{A}_{0}-{A}_{1}}{{A}_{0}} \times 100 \end{eqnarray*}

where A_0_ is the control absorbance, and A_1_ is the absorbance of CF, Vitamin C or EGCG. EC_50_ was calculated as µg/ml using sigmoid non-linear regression.

### *Caenorhabditis elegans* strains and maintenance

*C. elegans* strains used in this study are: Wild type N2, TJ356 (zIs356[daf-16p::daf-16a/b::GFP + rol-6]), CF1553 (mu1s84[pAD76(sod-3::GFP)]), TJ375 (gpIs1[hsp-16.2::GFP]), CL2166 (dvIs19[pAF15(gst-4::GFP::NLS)]), AM141 (rmls133[unc-54p::Q40::YFP]), CL4176 (dvIs27[myo-3p::A-Beta (1–42)::let-851 3′UTR) + rol-6(su1006)]) X and CL802 [smg-1(cc546) I; rol-6(su1006) II]. Nematodes were all cultured in nematode growth medium (NGM) containing *E. coli* OP50 as food source and kept at 20 °C incubator, except for CL4176 and CL802 which were kept at 16 °C. All strains and *E. coli* OP50 were obtained from Caenorhabditis Genetics center (CGC), University of Minnesota, U.S.A.

Age synchronization of worms was achieved by isolating eggs from gravid hermaphrodites using 5 M NaOH, 5% NaOCl (1:3) and sterile water, vortexing 10 min for lysis, followed by centrifugation at 1,200 rpm for 1 min. The supernatant was removed till 0.1 ml then sterile water was added till 5 ml and centrifugation was repeated at 1,200 rpm for 1 min. Finally, water was removed and eggs were kept in M9 buffer for hatching. Larvae were then kept after hatching in S-medium containing *E. coli* OP50 (OD_600_ = 1.0) (Stiernagle, 1999). Different treatments were applied according to each experiment.

### Survival assay under juglone–induced oxidative stress

Synchronized N2 worms (L1 larvae stage) were kept in S-medium at 20 °C using *E. coli* OP50 as food source. They were divided into six groups of 80 worms each, on the day after hatching, and treated differently. First group was left untreated (untreated control group). Second group was treated with 2.1% methanol (solvent control group). This group served to exclude any toxicity of the solvent used for dissolving the extracts on worms. Third group was treated with EGCG 50 µg/ml dissolved in water, used as a positive control for its known antioxidant activity (Abbas & Wink, 2009; Abbas & Wink, 2010). Groups four, five and six were treated with 100, 200 and 300 µg/ml CF dissolved in methanol (maximum 2.1%).

After 48 h of treatment, the pro-oxidant juglone was added at a lethal concentration (80 µM) for 24 h. Alive and dead worms were counted, considering the dead ones as those who did not move upon touching them gently and repetitively with a platinum wire. Results from three repetitions were calculated as mean ± SEM of live worms (in %). Comparison was done using one-way ANOVA then Bonferroni’s (post-hoc) method using Graphpad Prism version 5.01 (Abbas & Wink, 2009).

### Intracellular ROS levels

Age synchronized N2 worms (L1 stage) grown in S-medium with *E. coli* OP50 at 20 °C were divided into six groups, on the day after hatching. First group was left untreated; second one received 2.1% methanol and the third group was treated with 50 µg/ml EGCG. The other three groups were treated with 100, 200 and 300 µg/ml CF respectively. All groups were left for 48 h then washed with M9 buffer. Afterwards, 1 ml 50 µM of H_2_DCF-DA was added and samples were incubated for one hour away from light at 20 °C. Then worms were washed with M9 buffer to remove extra dye and mounted on a glass slide, with the addition of 10 mM sodium azide for paralysis induction.

Analysis was done by fluorescence microscope BIOREVO BZ-9000 with mercury lamp (Keyence Deutschland GmbH, Neu-Isenburg, Germany) at *λ*_ex_ 480/20 nm, *λ*em 510/38 nm. 30 worms, at least, were photographed from each group using 10X objective lens and constant exposure time. ImageJ software version 1.48 (National Institutes of Health, Bethesda, MD, USA) was then used to measure densitometrically the relative fluorescence of the full body. Results of three independent assays are expressed as mean fluorescence intensity and compared using one-way ANOVA then Bonferroni’s (post-hoc) method (Peixoto et al., 2016b).

### DAF-16 distribution

L1 age synchronized TJ356 transgenic worms, expressing DAF-16::GFP fusion protein, were grown in S-medium supplied with *E. coli* OP50 at 20 °C. On the day after hatching, they were divided into six groups and were treated as described previously. After 24 h, worms were mounted on a glass slide using 10 mM sodium azide for paralysis. Thirty different worms, at least, were visualized via 20X objective lens at constant exposure time. DAF 16::GFP fusion protein was located either in the nucleus or cytoplasm, whereas some worms showed intermediate distribution between the nucleus and cytoplasm. Analysis was done for three different runs as previously described. Worms were sorted and counted according to the location of the DAF-16::GFP to cytoplasmic, intermediate or nuclear (Peixoto et al., 2016a).

### SOD-3 expression

Synchronized CF1553 transgenic worms (L1 stage), expressing SOD-3::GFP as a reporter fusion protein, were grown in S-medium inoculated with *E. coli* OP50 at 20 °C. They were divided into six groups, on day one after hatching, and treated as previously described, except for the EGCG group that was treated with 100 µg/ml. After 72 h of treatment, worms were mounted on a glass slide with 10 mM sodium azide and images of 30 worms were taken at constant exposure time by 10X objective lens. Analysis of three runs was performed by measuring the mean fluorescence intensity of the posterior intestine and results were obtained as described before (Peixoto et al., 2016a).

### HSP-16.2 expression under juglone induced oxidative stress

TJ375 transgenic L1 worms expressing HSP-16.2::GFP reporter gene were divided into six groups, treated as previously mentioned on day one after hatching, and kept at 20 °C. After 72 h of treatment, the worms were exposed to a non-lethal dose of 20 µM juglone for 24 h (diluted immediately prior to the assay in M9 buffer from a 10 mM stock solution prepared in 99% ethanol) (Abbas & Wink, 2009). Worms were mounted on a glass slide with 10 mM sodium azide and images of at least 20 worms were taken at constant exposure time by 20X objective lens. Analysis of three runs was performed by measuring the mean fluorescence intensity of pharynx and results were obtained as described before.

### GST-4 expression

L1 larvae of synchronized CL2166 worms expressing GST-4::GFP reporter gene were kept in S-medium with *E. coli* OP50 at 20 °C. They were divided into six groups and treated as previously mentioned. Worms were kept for 48 h at 20 °C. Afterwards, worms were paralyzed with 10 mM sodium azide on a glass slide and 30 worms were imaged using 10X objective lens at constant time of exposure. Mean fluorescence intensity of the whole body was measured. The experiment was repeated three times and analysis was done as previously mentioned.

### PolyQ40 aggregates quantitative determination

L1, age synchronized transgenic AM141 worms expressing polyQ40::YFP as a reporter gene were cultured at 20 °C in S-medium containing *E. coli* OP50. All worms were divided into six groups and were treated for 72 h as mentioned before. Worms were then paralyzed on a glass slide using 10 mM sodium azide. Images were captured for at least 30 worms using 10X objective lens at constant exposure time. PolyQ40 aggregates, present in the muscle cells of the worms, were counted manually by visualizing the worms of three different assays. The results were expressed as mean ± SEM and compared using one-way ANOVA followed by Bonferroni’s (post-hoc) method.

### Paralysis assay

CL4176, temperature-inducible nematodes expressing Aβ transgene in their muscle cells were used in this assay to evaluate paralysis as a symptom of Aβ toxicity. CL802 nematodes, lacking Aβ transgene, were used as a control strain for testing effects independent from Aβ formation. CL4176 population was divided into 5 groups, 50–70 worms each. First group was left untreated (untreated control). Second group, solvent control was treated with 2.1% MeOH. This group was done to exclude any toxicity or paralysis delaying effect of the solvent used to dissolve the extracts. EGCG group 100 µg/ml was used as a positive control and the last two groups were given CF (300 and 500 µg/ml respectively). The assay was performed as described by Dostal & Link (2010), where treatments were added to separate NGM plates and left in 16 °C incubator for 24 h. Afterwards, *E. coli* OP50 was added in the middle of the plate and plates were left again for 24 h at 16 °C. Then bleaching was done, to obtain age-synchronized population, and 50–70 eggs were put in the middle of the plate. All plates were kept for 48 h at 16 °C then an automatic upshift was done to 25 °C to induce Aβ transgene expression.

Paralysis was assayed 22 h after the upshift and L4 stage worms were observed every 1–2 h for 12 h. Paralyzed worms were the ones who did not respond to a gentle touch with a platinum wire. Three independent assays were carried out and the data is expressed as mean ± SEM. Analysis was done using Kaplan–Meier survival analysis to get the time where half worms were paralyzed (PT_50_) and survival analysis was done to obtain the graph using a log-rank test in Graphpad prism, version 7.04. The results were compared using one-way ANOVA followed by Bonferroni’s method (post-hoc).

## Results

### LC/MS metabolites profiling

**Figure 1:**
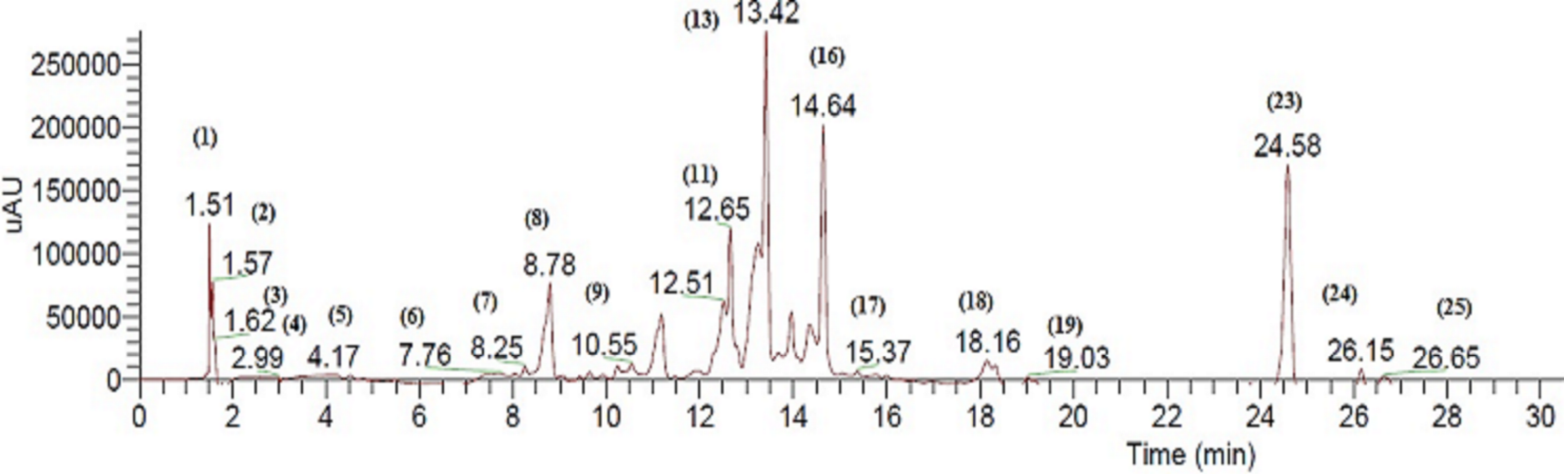
Negative LC/ESI/mass chromatogram of metabolites detected in a hydroalcoholic extract of *C. fistula*.

The bioactive extract of *C. fistula* was analyzed by HPLC, in combination with a mass spectrometry detector. ESI/LC/MS analysis was performed using cone voltage providing useful additional fragmentation data. A representative UV chromatogram is shown in Fig. 1. Secondary metabolites were tentatively identified, based on authentic standards when available, or on retention data as reported in Table 1, by ESI mass spectra and in comparison with the literature.

**Table 1:** Peaks assignment for metabolites detected in the bioactive hydroalcoholic extract of *Cassia fistula* L., using HPLC/PDA/ESI-MS^n^.

Peak #	Tentatively identified compounds	Retention time (min)	UV-Vis (*λ*max)	[M-H]^−^ (m/z)	Fragment ions (m/z)	Reference
*Cf1*	Galloylquinic acid^a^	1.51	274	343.04	191, 169, 152	Wyrepkowski et al. (2014)
*Cf2*	Quinic acid^a^	1.57	275	191.02	191	Handoussa et al. (2013)
*Cf3*	Galloyl glucose derivative	1.62	273	479.08	481, 425, 301, 299	Mena et al. (2012)
*Cf4*	Acetyl-p-coumaroyl- caffeoylglycerol^a^	2.99	320	441.14	381, 295, 179, 163, 135	Bertrams et al. (2013)
*Cf5*	Acetyl-dicaffeoylglycerol^a^	4.17	292, 318	457.13	397, 295, 235, 161, 135	Bertrams et al. (2013)
*Cf6*	Quercetin-caffeoylglucoside^a^	7.76	253, 348	625.1	462, 301, 300	Francescato et al. (2013)
*Cf7*	Galloyl glucose derivative	8.25	273	479.08	481, 425, 301, 299	Mena et al. (2012)
*Cf8*	Quercetin hexoside^a^	8.78	256, 358	463.5	301, 179	Handoussa et al. (2013)
*Cf9*	Kaempferol 3-*O*-[6^′′^-*O*-(rhamnosyl) glucoside]7-*O*-rhamnoside	10.55	348, 290	739.34	284	Schatz, Lapierr & Ralph (2003)
*Cf10*	Kaempferol-*O*-coumaryl-*O*-glucoside	11.4	327	593.3	429, 284	Brito et al. (2014)
*Cf11*	Sennoside B	12.65	335	861.17	699, 537, 431, 385	Std.
*Cf12*	Eriodictyol-7-*O*-neohesperidoside^a^	13.2	326	594.9	449, 287	Brito et al. (2014)
*Cf13*	Rhein-*O*-glucoside	13.4	325	445.04	283, 257, 239	Ye et al. (2007)
*Cf14*	(Iso)rhamnetin 3-*O*-glucoside-7-*O-* rhamnoside^a^	14.1	290, 348	623.08	477, 383, 315, 314	Li et al. (2016)
*Cf15*	Isorhamnetin-3*-O-* rhamnoside^a^	14.2	250, 342	461.1	315, 301	Brito et al. (2014)
*Cf16*	Procyanidin dimer	14.6	285	579.5	425, 407	Venter, Joubert & De Beer (2013)
*Cf17*	Quercetin deoxyhexoside^a^	15.3	249, 361	447.09	301, 179	Silva et al. (2014)
*Cf18*	Quercetin acetyl hexoside^a^	18.16	254, 354	505.10	301, 179	Barros et al. (2012)
*Cf19*	Quercetin-*O*-(*O*-galloyl)-hexoside^a^	19.03	254, 356	615.21	463, 301, 271	Saldanha, Vilegas & Dokkedal (2013)
*Cf20*	Luteolin 7-*O*-rutinoside^a^	22.6	254, 267, 335	593.15	447, 284	Brito et al. (2014)
*Cf21*	Kaempferol-3-*O*-acetylglucoside	22.7	352, 266	489.10	285	Kajdzanoska, Gjamovski & Stefova (2010)
*Cf22*	Luteolin-7-*O*-(6^′′^-quinoyl)-rhamnosyl-6-C-pentosyl-8-C,*O*-(6^′′′^acetyl)-glucoside^a^	23.12	270, 344	925.30	605, 563, 443	Benayad, Gomez-Cordoves & Es-Safi (2014)
*Cf23*	Sennoside aglycone.	24.58	254,365	537.5	268	Ye et al. (2007)
*Cf24*	Kaempferol 3-*O*-[6^′′^-*O*-(rhamnosyl) glucoside] 7-*O*-rhamnoside	26.15	326	739.34	593,284	Schatz, Lapierr & Ralph (2003)
*Cf25*	Sweroside^a^	26.65	215,325	403.12	179	Lin et al. (2015)
*Cf26*	Procyanidin monogallate derivative	31	277, 236	891.01	695, 577, 575	Rockenbach et al. (2012)
*Cf27*	Proanthocyanidin derivative	33.5	278, 234	525.16	407, 289	Kajdzanoska, Gjamovski & Stefova (2010)
*Cf28*	Coumaric acid glucoside dimer^a^	33.9	275, 326	651.05	325, 163	Simirgiotis et al. (2015)
*Cf29*	Decarboxylated rhein derivative	34.2	310	239.34	211	Fuh & Lin (2003)
*Cf30*	Rhein derivative	34.8	310	257.11	239	Ye et al. (2007)
*Cf31*	Emodin-*O*-glucoside	35.2	312	431.5	269, 224	Ye et al. (2007)
*Cf32*	Isoaloeresin D^a^	35.8	317	555.17	538, 512, 410	El Sayed et al. (2016)

**Notes.**

a Compounds identified for the first time from *C. fistula*.

Quinic acid derivatives were detected as galloyl quinic acid, represented by peak *Cf1* which has [M–H]^−^ of m/z 343.04. Furthermore, quinic acid as in peak *Cf2* with its deprotonated molecule were detected as [M–H–152]^−^ at m/z 191.02. This was due to elimination of a galloyl group. The corresponding fragmentation at m/z 169 was also detected, which explains the elimination of quinic acid and the formation of deprotonated gallic acid (Wyrepkowski et al., 2014).

Several glycosides were identified as major peaks within the bioactive extract, as peak *Cf9* which was identified as kaempferol 3-*O*-[6″-*O*-(rhamnosyl) glucoside] 7-*O*-rhamnoside as [M–H]^−^ at m/z 739 ([K + 2Rha + Glu–H]^−^ ). Another peak appeared at m/z 284, representing kaempferol aglycone (Schatz, Lapierr & Ralph, 2003). Furthermore, peak *Cf10* was identified as kaempferol-*O*-coumaroyl glucoside with m/z 593.3 as the base peak. The main fragment appeared at m/z 284. A difference of 308 indicates the loss of hydrated coumaroyl glucoside moiety (Brito et al., 2014). Kaempferol-related glycosides were previously reported within the same species (Bahorun, Neergheen & Aruoma, 2005).

In addition, various glycosides were identified, such as eriodictyol-7-*O*-neohesperidoside represented by peak *Cf12*, as [M–H]^−^ ion at m/z 594.9. Fragments appeared at m/z 449 and m/z 287, and (iso) rhamnetin-3-*O*-glucoside-7-*O*-rhamnoside represented by *Cf14*, which produced [M–H]^−^ at m/z 623.08. In the MS–MS spectrum, two predominant fragments at m/z 315, due to deprotonated aglycone [M–H–308]^−^ and 314, due to [M–H–308]^−•^ appeared. This indicates that a hexose (162) and a deoxyhexose (146) are linked at the same position of the aglycone. Due to the most abundant fragment at [M–H–308]^−•^, the substituent position is suggested to be a 3-OH group. As a result, the aglycone is identified as (iso) rhamnetin, reported previously in *C. fistula* (Bahorun, Neergheen & Aruoma, 2005).

*Cf5* was identified as acetyl-dicaffeoylglycerol producing an ion at m/z 457.13. Further fragments were observed at m/z 161 and 135. Furthermore, acetyl-p-coumaroyl-caffeoylglycerol, represented by peak *Cf4*, exhibited a deprotonated molecular ion [M–H]^−^ at m/z 441.

*Cf11* was identified as sennoside B with deprotonated ion [M–H]^−^ at m/z 861.17 and the fragmentation pattern showed m/z 699 due to the loss of one glucose molecule [M–H–glu]^−^ and m/z 537 due to the loss of 2 glucose molecules [M–H–glu–glu]^−^ (Zhao et al., 2012). It is worth noting that sennoside B was also compared to an authentic standard to prove its identity. Peak *Cf13* was identified as rhein-*O*-glucoside. Their MS/MS spectrum yielded m/z 283 as the base peak, which could further fragment into m/z 257 and m/z 239. These cleavages were similar to those of rhein. Therefore, the corresponding compound was tentatively identified as the known rhein-*O*-glucoside (Ye et al., 2007). Peak *Cf31* was tentatively identified as emodin-*O*-glucoside; its MS/MS spectrum showed ions at m/z 269 and m/z 268. The MS3 spectrum of m/z 268 gave a fragment at m/z 224, indicating the loss of CO_2_ [M–H–CO_2_]^−^, and demonstrating the emodin as aglycone, which has been previously detected as a major constituent in *C. fistula* (Lee, Lee & Kuo, 2001; Ye et al., 2007).

### Total phenolic content

In order to generally quantify the amount of phenolics in the extract, the Folin-Ciocalteu assay was carried out. It showed a total phenolic content of 93.79 ± 4.13 µg of GAE/mg of extract, a value that is close to those obtained from different type of extracts of the aerial parts from different *Cassia* species (Mehta et al., 2017).

### CUPRAC method for total antioxidant capacity

Cupric ion reducing antioxidant capacity assay (CUPRAC), is one of the assays based on electron transfer like DPPH, FRAP and Folin-Ciocalteu. It measures the ability of the antioxidant to reduce the oxidant, leading to a color development that could be measured spectrophotometrically. Polyphenolic compounds have distinctively reactive Ar-OH groups, which are able to be oxidized to their quinone analogue.

Consequently, a reduction to the chromogenic CUPRAC reagent, bis (neocuproine) copper (II) chelate to bis (neocuproine) copper (I) chelate takes place leading to development of yellow-orange color having a maximum absorption at 450 nm. Protons liberated from the reaction are buffered by ammonium acetate (Apak et al., 2004). A strong antioxidant capacity is represented by low EC_50_ value. BHA standard was used as a positive control in this study. CF showed a relatively close EC_50_value (EC_50_ = 170 ± 0.55 µg/ml) to BHA (Table 2).

**Table 2:** *In vitro* antioxidant activity of *C. fistula* extract using CUPRAC and DPPH assays.

Sample	CUPRAC^a^ (µg/ml)	DPPH^a^ (µg/ml)
CF	170 ± 0.55	52.6 ± 0.86
EGCG	–	1.4 ± 0.06
Vit C	–	2.7 ± 0.09
BHA	167 ± 0.52	–

**Notes.**

a EC_50_ in µg/ml.

### DPPH assay

CF showed a moderate antioxidant activity in DPPH assay (EC_50_ of 52.6 ± 0.86 µg/ml), compared to the positive controls Vit C and EGCG (Table 2).

### Survival assay

**Figure 2:**
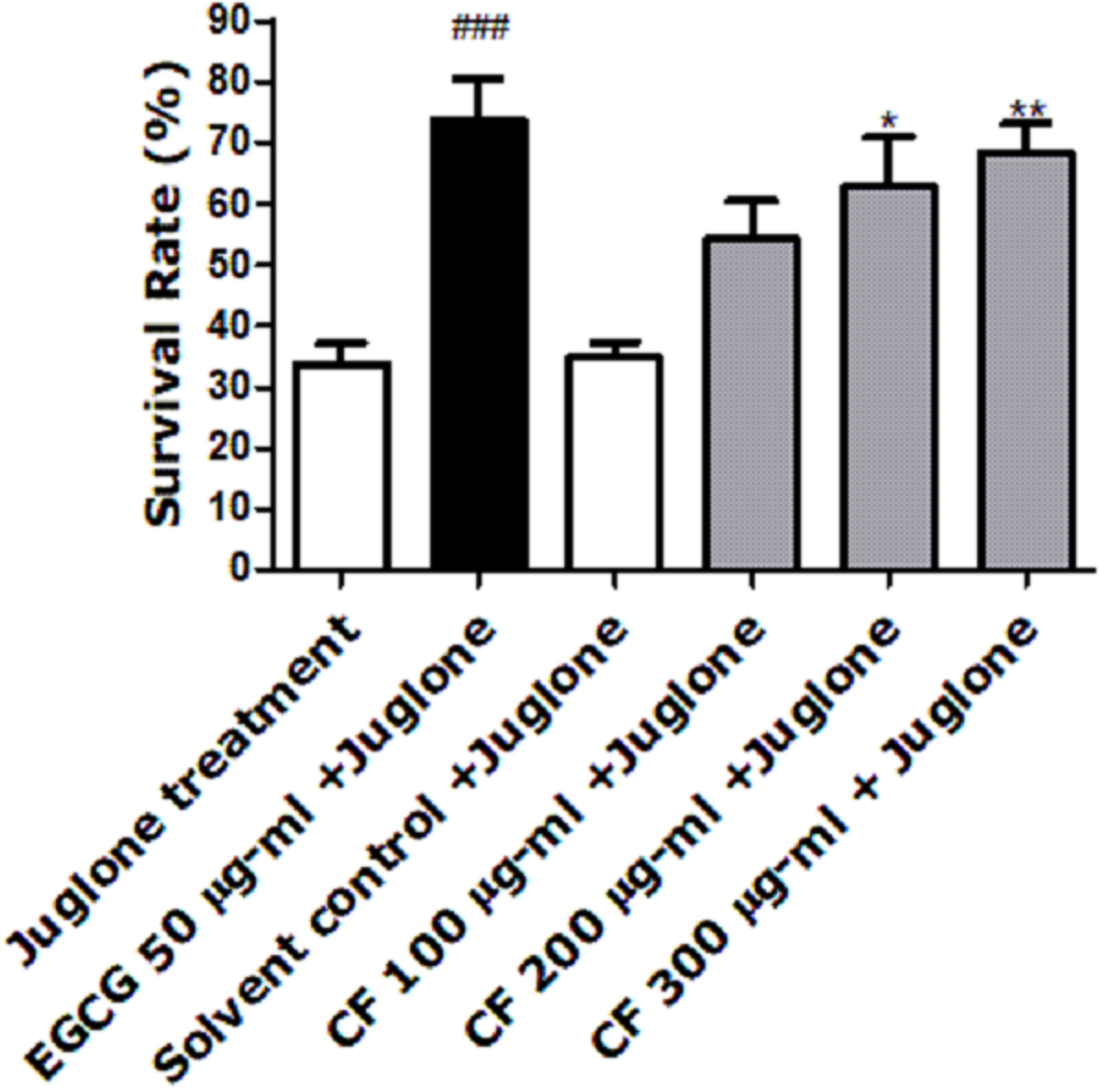
Effect of *C. fistula* extract on increasing stress resistance in *C. elegans* worms exposed to lethal dose of juglone. Survival rate of wild-type (N2) worms pre-treated with CF, followed by exposure to oxidative stress induced by juglone. CF 300 µg/ml + juglone group showed an enhancement in survival rate compared to juglone treated group. Results are presented as mean percentage of survivals ± SEM of three different runs using one-way ANOVA followed by Bonferroni’s method (post-hoc). *p < 0.05 and **p < 0.01 compared to solvent control + juglone group and ^###^*p* < 0.001 compared to juglone treated group.

Juglone, a yellow pigmented pro-oxidant quinone found in *Juglans regia*, is used at a high concentration to induce death of the nematode (Inbaraj & Chignell, 2004). Pre-treatment of worms with CFs enhanced survival rates. CF 300 µg/ml showed the highest percentage (68 ± 5.14%) when compared to the solvent control, MeOH, showing only 34 ± 2.53% survival (*p*-value <0.001), (Fig. 2). These results are close to the EGCG group, positive control known for its powerful antioxidant activity, which showed a 73 ± 6.73% survival rate (Abbas & Wink, 2009).

### Effect of CF on intracellular ROS levels

To further test the antioxidant effect of CF *in vivo*, intracellular ROS levels were evaluated using wild type N2 worms. H_2_DCFDA, a membrane permeable compound, is a widely known marker for detecting the production of ROS inside the cell. After entering the cell, the compound is deacetylated by the intracellular esterases. The presence of ROS leads to the oxidation of the compound forming the highly fluorescent 2′,7′-dichlorofluorescin, where the intensity of the fluorescence correlates with the levels of ROS (Eruslanov & Kusmartsev, 2010).

**Figure 3:**
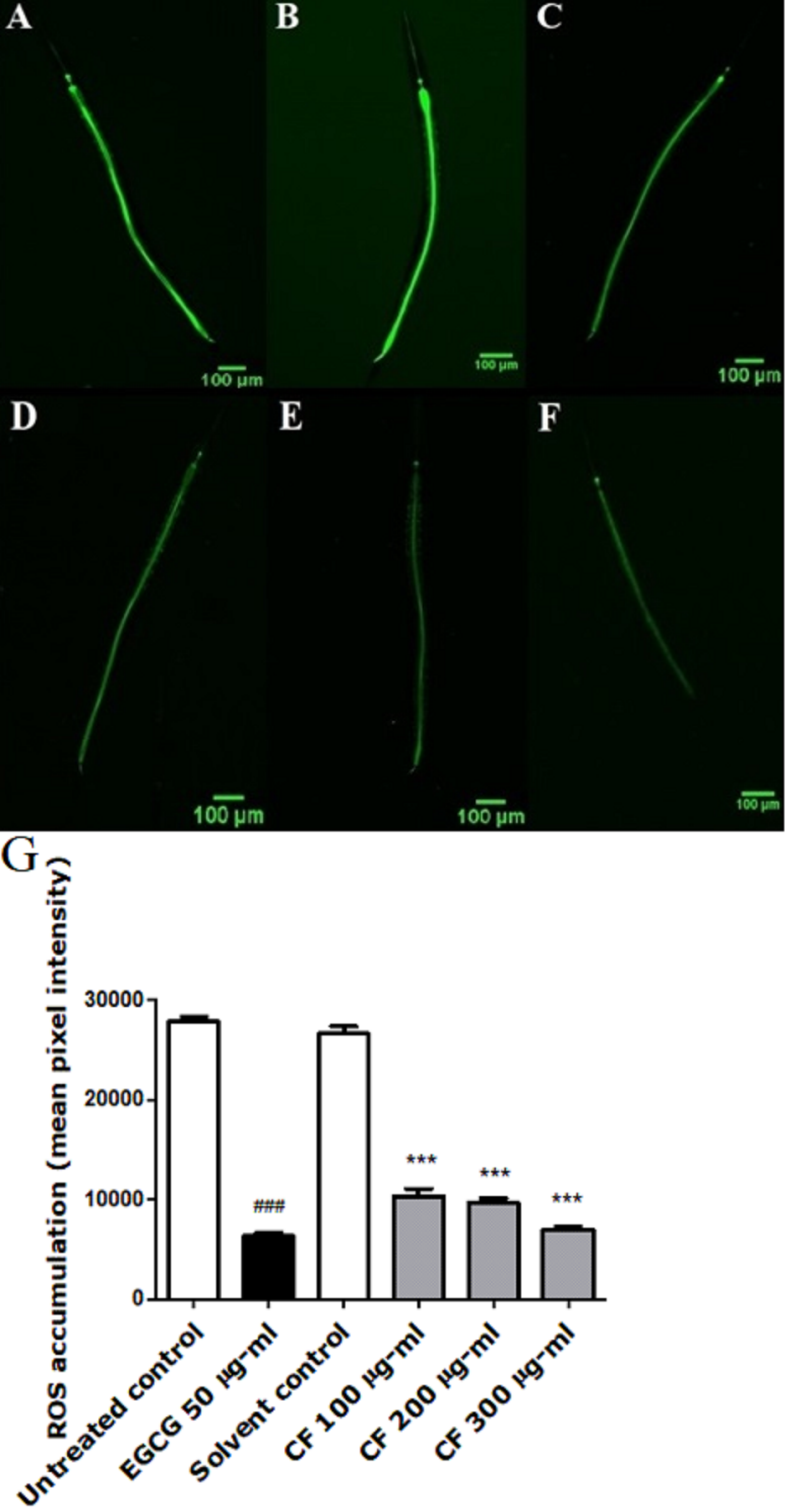
Effect of *C. fistula* extract on intracellular ROS levels in *C. elegans*. (A) Untreated control. (B) Solvent control. (C) CF 100 µg/ml. (D) CF 200 µg/ml. (E) CF 300 µg/ml. (F) EGCG 50 µg/ml. (G) Intracellular accumulation of ROS in N2 worms. CF 100, 200 and 300 µg/ml significantly decreased the levels of ROS compared to the solvent control group. Data is presented as mean of three different runs using one-way ANOVA followed by Bonferroni’s method (post-hoc). ***p < 0.001 compared to solvent control group and ^###^*p* < 0.001 compared to untreated control group.

Results show a significant concentration dependent decrease in the fluorescent intensity of the extract treated group, up to 73% decrease in CF 300 µg/ml compared to MeOH group. These results are in accordance with the EGCG group, which showed 77% decrease in fluorescence compared to the untreated control (*p*-value <0.001) (Fig. 3).

### Effect of CF on DAF-16 localization

To detect whether the antioxidant effect of CF is based only on direct ROS scavenging activity or if other mechanisms are involved, DAF-16 assay was carried out. The DAF-16 transcription factor of *C. elegans* is considered as a homologue to the fork head transcription factor (FOXO), found in humans. Normally, DAF-16 is localized in the cytosol in its inactive phosphorylated form. Certain kinds of triggers, like stress and other environmental factors can induce its dephosphorylation and subsequent nuclear localization.

**Figure 4:**
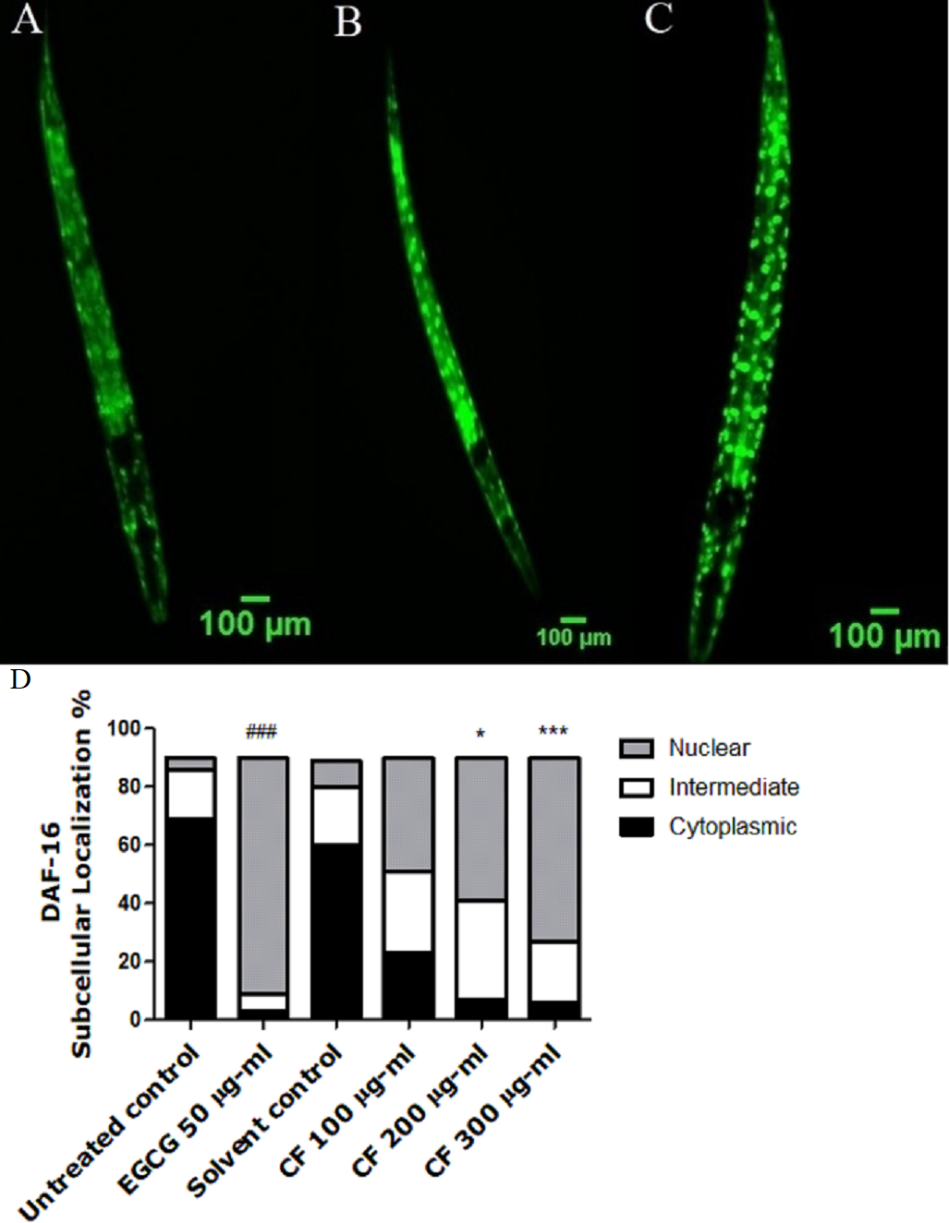
Effect of *C. fistula* extract on DAF-16 localization in *C. elegans*. (A) Cytoplasmic localization. (B) Intermediate localization. (C) Nuclear localization of DAF-16 transcription factor. (D) Results show the percentage of worms showing cytosolic, intermediate and nuclear localization of DAF-16. CF 300 µg/ml increases the translocation of DAF-16::GFP in TJ356 mutant worms to the nucleus. Data are shown as mean ± SEM for three different experiments analyzed by one-way ANOVA then Bonferroni (post-hoc). *p < 0.05, ***p < 0.001 compared to solvent control group and ^###^*p* < 0.001 compared to untreated control group.

Upon nuclear localization, DAF-16 activates several genes that are responsible for lifespan extension and stress response (Mukhopadhyay, Oh & Tissenbaum, 2006). TJ356 transgenic worms are used in this assay in which the treatment with CF 300 µg/ml clearly showed high nuclear localization of DAF-16::GFP by 63 ± 1.73%, compared to MeOH group with 8 ± 8% nuclear localization. On the other side, EGCG treated group showed 81 ± 6.24% nuclear localization (p-value <0.001). The previous results highly imply that the *in vivo* antioxidant effect of CF is mediated through DAF-16/FOXO pathway (Fig. 4).

### Effect of CF on SOD-3 expression

DAF-16 activation subsequently results in activating other stress response genes like SOD-3, which encodes mitochondrial Mn-SOD. The SOD-3 enzyme protects worms from ROS via scavenging O${}_{2}^{.-}$ radical (Honda & Honda, 1999). Antioxidant compounds show a high capability to increase the expression of SOD-3 (Zhou, Pincus & Slack, 2011). EGCG group showed 83% increase in fluorescence intensity compared to the untreated control.

**Figure 5:**
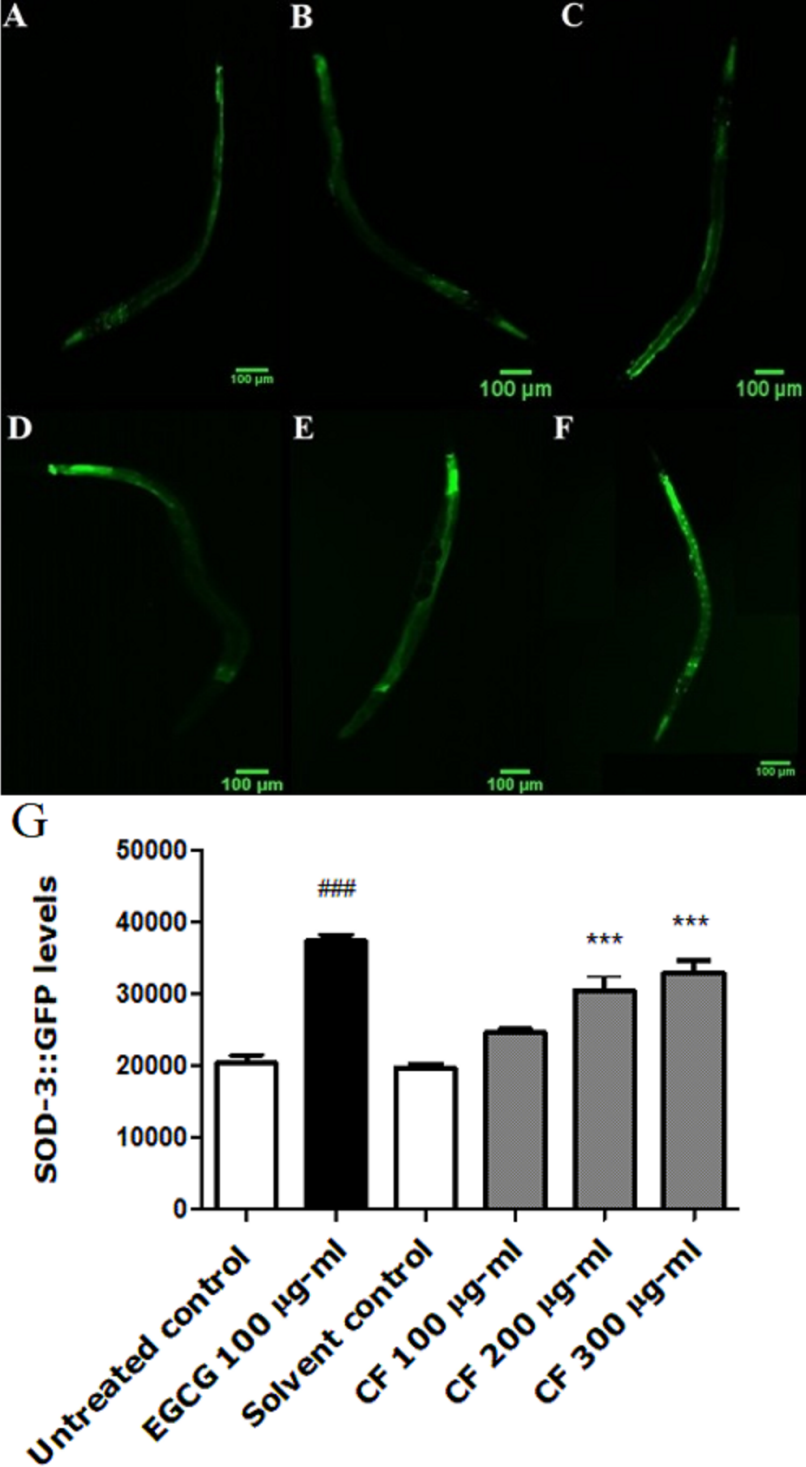
Effect of *C. fistula* extract on SOD-3 expression in *C. elegans*. (A) Untreated control. (B) Solvent control. (C) CF 100 µg/ml. (D) CF 200 µg/ml. (E) CF 300 µg/ml. (F) EGCG 100 µg/ml. (G) SOD-3 levels through quantification of GFP expression. CF 200 and 300 µg/ml increase the expression of SOD-3::GFP in CF1553 mutant worms compared to solvent control group. Shown results are represented as mean of three different experiments analyzed by one-way ANOVA followed by Bonferroni (post-hoc). ***p < 0.001 compared to solvent control group and ^###^*p* < 0.001 compared to untreated control group.

CF-treated worms show a higher expression of the SOD-3::GFP compared to the negative control. CF 300 µg/ml showed 67% increase in fluorescence intensity compared to MeOH group (*p*-value <0.001) (Fig. 5).

### Effect of CF on HSP expression

**Figure 6:**
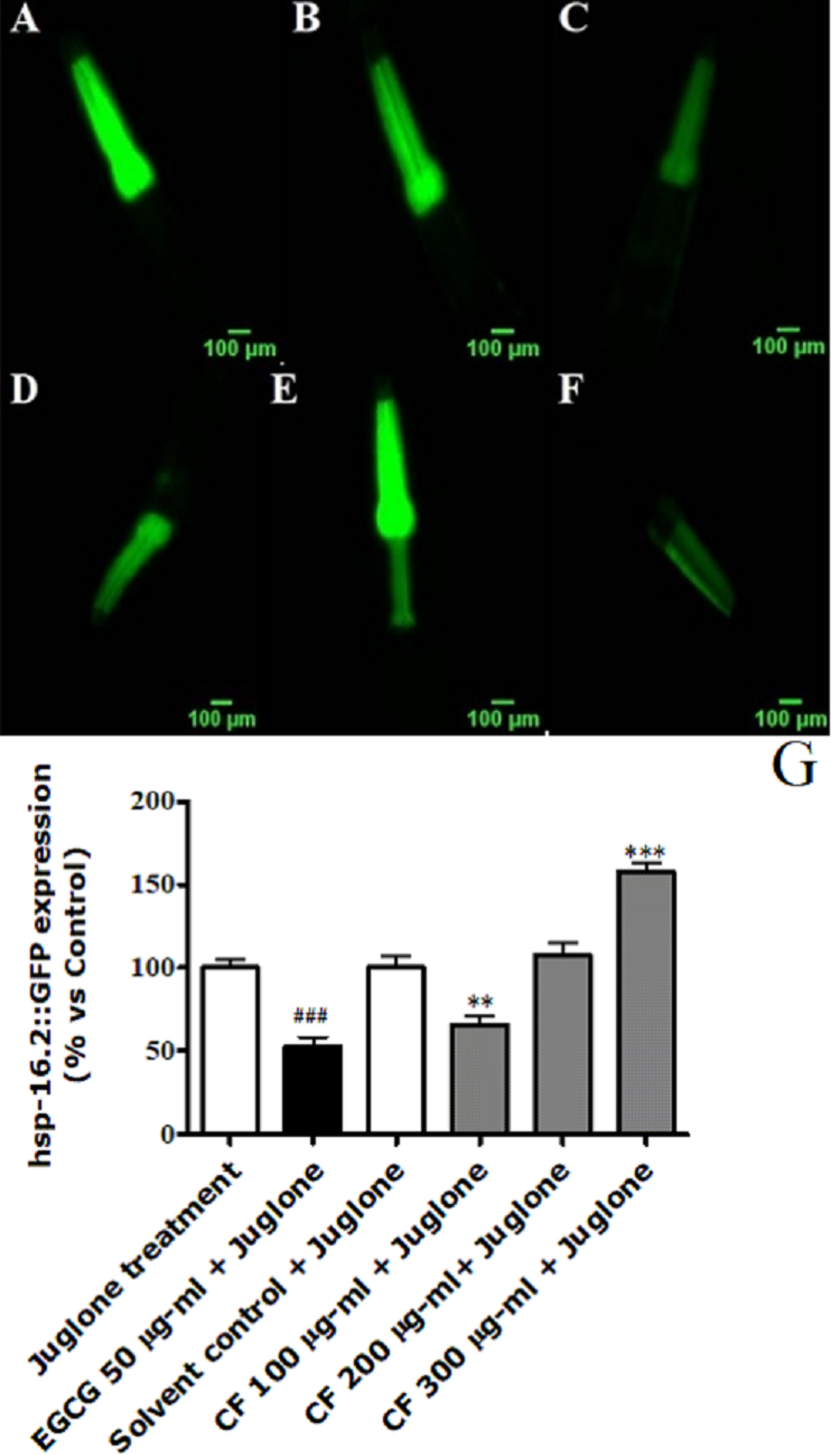
Effect of *C. fistula* extract on HSP-16.2 expression in *C. elegans* worms exposed to oxidative stress induced by juglone. (A) Juglone treated group. (B) Solvent control + juglone. (C) CF 100 µg/ml + juglone. (D) CF 200 µg/ml + juglone. (E) CF 300 µg/ml + juglone. (F) EGCG 50 µg/ml + juglone. (G) HSP-16.2 levels through quantification of GFP expression. CF 100 µg/ml + juglone decreased the expression of HSP-16.2::GFP in TJ375 mutant worms compared to solvent control + juglone group. CF 300 µg/ml + juglone increased the expression of HSP-16.2 compared to solvent control + juglone group. Shown results are analyzed by one-way ANOVA followed by Bonferroni (post-hoc). **p < 0.01, ***p < 0.001 compared to solvent control + juglone group and ^###^*p* < 0.001 compared to juglone treated group.

To further support the antioxidant effect of the extract, quantification of heat shock protein (HSP) expression in TJ375 mutant worms was investigated. HSP-16.2 is used to test stress levels in worms, as it is induced by both oxidative stress and heat (Strayer et al., 2003). The lowest concentration tested of the extract, CF 100 µg/ml, showed a lower expression of HSP-16.2, induced by juglone (34.34 ± 5.59%) when compared to solvent control + juglone treated group. EGCG showed a reduction in the fluorescence intensity with a value of 47.45 ± 5.67%, compared to juglone treated group (*p*-value <0.001) (Fig. 6).

### Effect of CF on GST-4 expression

**Figure 7:**
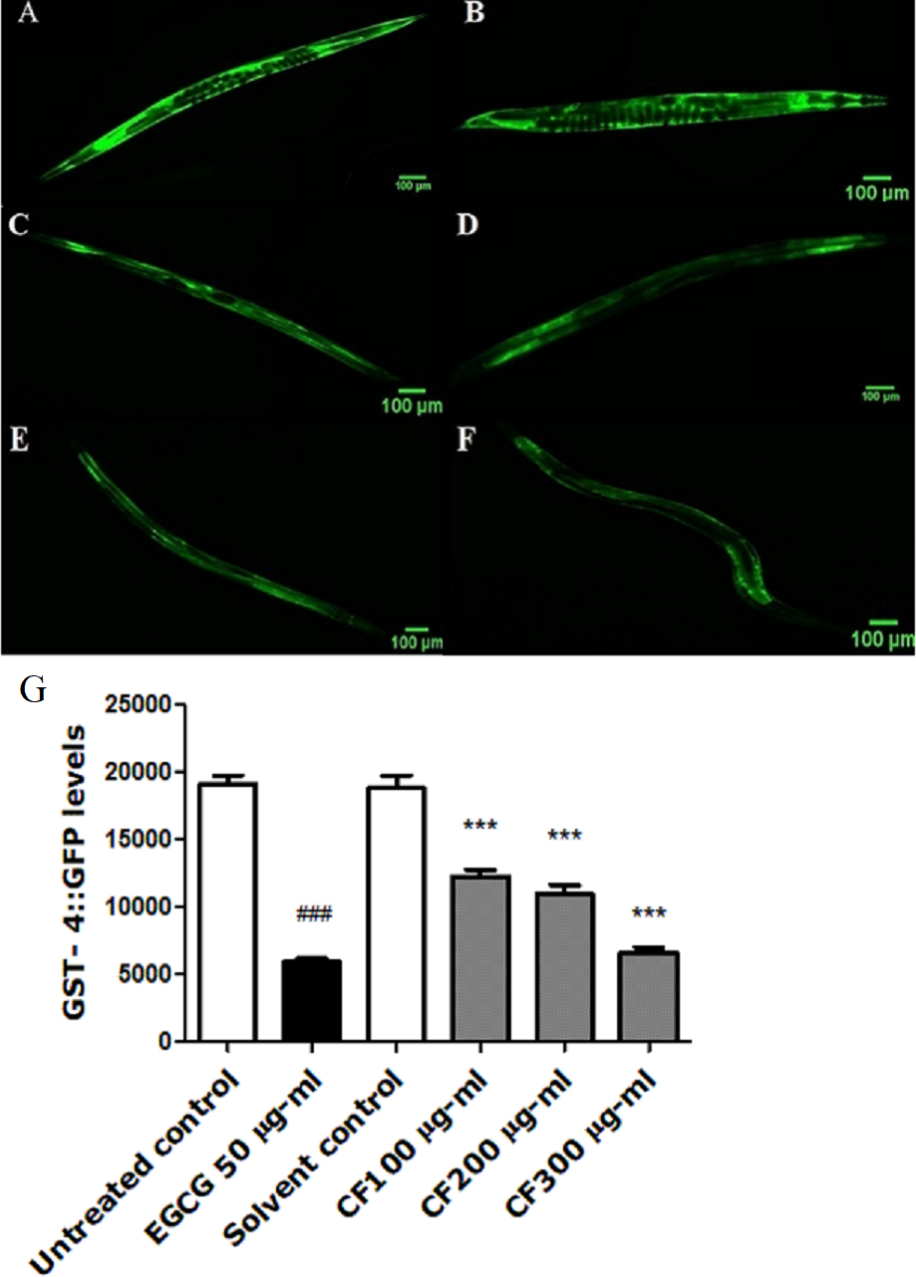
Effect of *C. fistula* extract on GST-4 expression in *C. elegans*. (A) Untreated control. (B) Solvent control. (C) CF 100 µg/ml. (D) CF 200 µg/ml. (E) CF 300 µg/ml. (F) EGCG 50 µg/ml. (G) GST-4 levels through quantification of GFP expression. CF 100, 200 and 300 µg/ml decreased the expression of GST-4::GFP in CL2166 mutant worms compared to solvent control group. Results are represented as mean of three different runs analyzed by one-way ANOVA and Bonferroni (post-hoc). ***p < 0.001 compared to solvent control group and ^###^*p* < 0.001 compared to untreated control group.

GST-4 is one of the glutathione S-transferases contributing to Phase II detoxification process (Choe, Przybysz & Strange, 2009). This group of enzymes is involved in the response towards oxidative stress. They are regulated by the SKN-1 transcription factor, a homologue to the human NRF2, which targets downstream genes (Kahn et al., 2008). Results show a marked decrease in the expression of GST-4 in worms treated by both EGCG and CF, compared to the negative control groups. CF showed 65% decrease in fluorescence intensity compared to MeOH group. These results are in accordance with EGCG treated group that showed 69% decrease in fluorescence intensity compared to the negative control (*p*-value <0.001) (Fig. 7).

### Effect of CF on PolyQ40 aggregation

**Figure 8:**
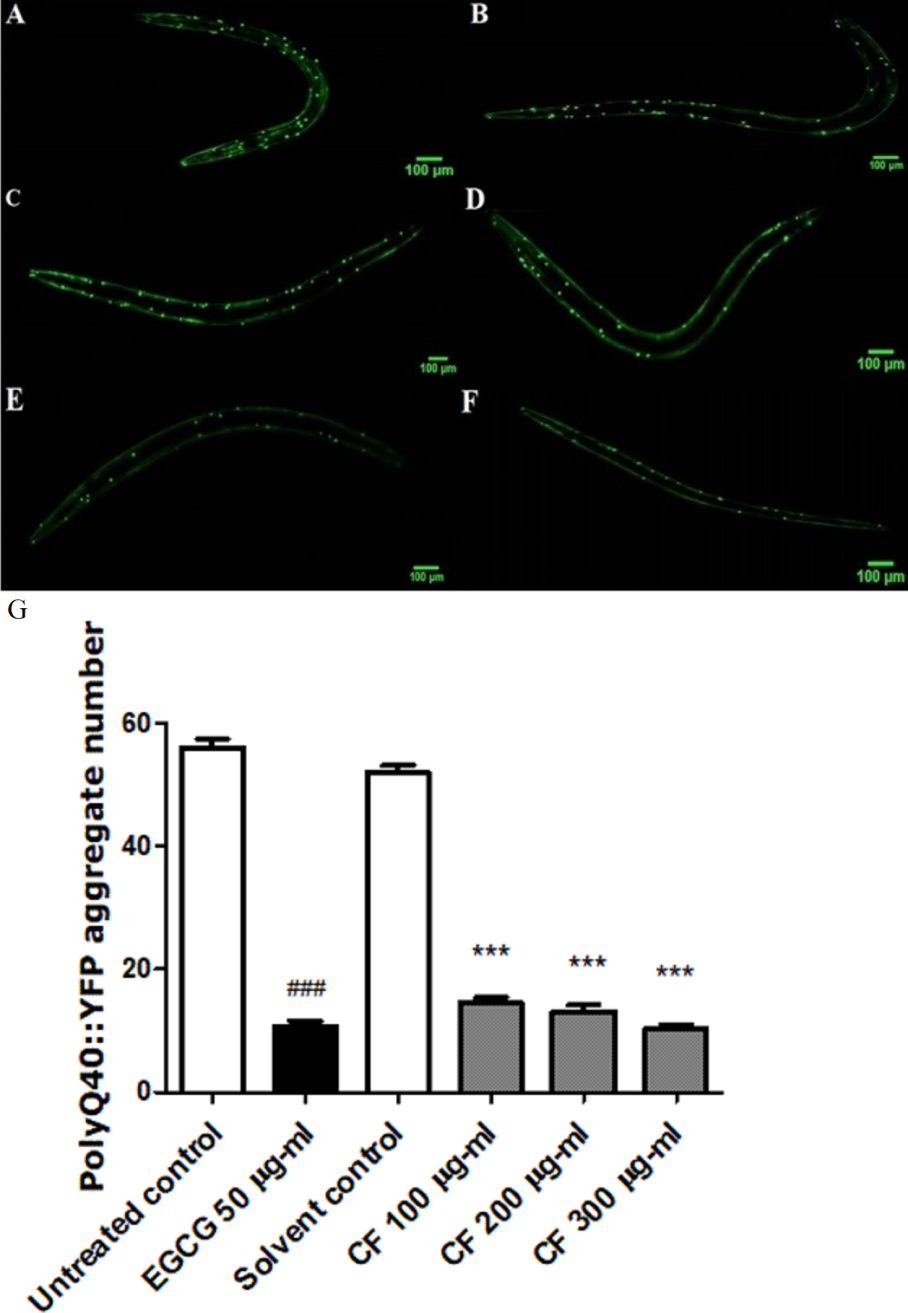
Effect of *C. fistula* extract on polyQ40 aggregate formation in *C. elegans*. (A) Untreated control. (B) Solvent control. (C) CF 100 µg/ml. (D) CF 200 µg/ml. (E) CF 300 µg/ml. (F) EGCG 50 µg/ml. (G) Accumulation of polyQ40::YFP aggregates in AM141 mutant worms. CF 100, 200 and 300 µg/ml treated worms showed a massive decrease in aggregates number compared to solvent control group. Data is represented as mean ± SEM for three experiments analyzed by one-way ANOVA and Bonferroni (post-hoc). ***p < 0.001 compared to solvent control group and ^###^*p* < 0.001 compared to untreated control group.

PolyQ40 aggregation is linked to Huntington’s disease and other neurodegenerative diseases (Williams & Paulson, 2008). Results in this study show that treatment with 300 µg/ml CF is able to decrease the number of polyQ40 aggregates by 80 ± 0.64% compared to MeOH group. This result matches with the positive control, EGCG treated group thaat also showed 80 ± 0.83% decrease in aggregate formation compared to the control group (*p*-value <0.001). This data demonstrates the strong influence of CF in attenuating protein aggregation (Fig. 8).

### Effect of CF in A**β** induced paralysis

To further support the neuroprotective effect of CF, a paralysis assay was carried out. The CL4176 strain was used to test the effect of the plant extract on Aβ accumulation and toxicity, that result in worm paralysis. Results are expressed as a delay in PT_50_, time where 50% of the worms are paralyzed, compared to the control group. Three hundred µg/ml CF showed a 4 h delay in paralysis time while CF 500 µg/ml showed a 5 h delay, a result that is close to that of the positive control, 100 µg/ml EGCG, used (*p*-value <0.0001) (Table 3). CL802 control strain, showed no paralysis at all, regardless of treatment.

**Table 3:** Median paralysis time, PT_50_, in *C. elegans* strain CL4176 using different concentrations of *C. fistula* extract; *p* values are calculated in comparison to either the untreated or the solvent control.

Treatment	PT_50_ ± SEM (h)	Significance
Untreated control	26.33 ± 0.33	
EGCG 100 µg/ml	33.33 ± 0.33	*P* < 0.0001
Solvent control	27.00 ± 0.00	
CF 300 µg/ml	31.33 ± 0.33	*P* < 0.0001
CF 500 µg/ml	32.00 ± 0.57	*P* < 0.0001

## Discussion

Free radicals are known for their deleterious effects and involvement in many human diseases. Natural products like Green tea, blue berries, ginger and *Ginkgo biloba* have been extensively used in medicine for their pronounced antioxidant and neuroprotective activities (Strayer et al., 2003; Andres-Lacueva et al., 2005; Abbas & Wink, 2009; Abbas & Wink, 2010; Rahmani, 2014). These activities are attributed to the presence of flavonoids, catechins, anthocyanins and other polyphenolic compounds which are known for their ability to scavenge free radicals and to modulate expression of genes that counteract oxidative stress *in vivo* (Kampkotter et al., 2007a; Kampkötter et al., 2007b). *C. fistula* is a traditional medicinal plant that is known for its laxative, antipyretic, analgesic, anti-inflammatory, antifungal, antibacterial and antioxidant properties (Patel et al., 1965; Satyavati, Raina & Sharma, 1976; Ramakrishna-Rao & Gupta, 1977; Manonmani et al., 2005; Ilavarasan, Malika & Venkataraman, 2006).

The potency of CF hydroalcoholic extract demonstrated by its antioxidant and neuroprotective effects in *C. elegans*, might be attributed to its wide array of phytoconstituents.

LC/MS showed several peaks, *Cf6*, *Cf8*, *Cf17*, *Cf18* and *Cf19*, of quercetin derivatives in CF extract. This confirms the presence of quercetin aglycone in CF, and it is believed to have a role in the antioxidant activity shown by the extract. Quercetin is a known antioxidant, studies revealed its ability to attenuate the levels of ROS and decrease the degree of glutathione oxidation in *Saccharomyces cerevisiae* cells. It also showed neuroprotective activity, demonstrated by increasing chronological lifespan of yeast cells and enhancement of learning and memory in mice (Liu, Yu & Ning, 2006; Belinha et al., 2007). In addition, quercetin was able to increase stress resistance and life span of *C. elegans* through increasing DAF-16 translocation to the nucleus (Kampkötter et al., 2008).

Kaempferol glycosides are also prominent secondary metabolites found in CF extract, shown in peaks *Cf9*, *Cf10*, *Cf21* and *Cf24* where *Cf10* is tentatively identified as Kaempferol-*O*-coumaryl-*O*-glucoside. Kaempferol is believed to contribute to the bioactivity of the whole extract as it has previously showed strong antioxidant activity (Miean & Mohamed, 2001), while kaempferol conjugated to p-coumaroyl moiety shows considerable antioxidant and neuroinflammation inhibitory activity in comparison to similar structures that lack the coumaroyl moiety (Li et al., 2017). Kaempferol has also demonstrated antioxidant and neuroprotective effects in *C. elegans* model through enhancing the translocation of DAF-16 transcription factor from the cytosol to the nucleus (Kampkötter et al., 2007b).

The compounds emodin and rhein, *Cf31*, *Cf 13* and *Cf 30*, are not only powerful laxatives, but also free-radical scavengers with the potential to act as anticarcinogenic and neuroprotective agents (Cai et al., 2004; Vargas, Diaz & Carbonell, 2004). They might have a role in the neuroprotective and antioxidant effects of CF extract.

Additionally, several procyanidin derivatives were detected, *Cf16*, *Cf26* and *Cf27*, which could be participating in the overall effect of the extract due to their considerable antioxidant, anticancer, neuroprotective and radical scavenging activity (Koleckar et al., 2012; Wen et al., 2015).

The antioxidant effect was further confirmed by the *in vitro* DPPH and CUPRAC assays. CF showed close antioxidant effect to that of BHA standard in CUPRAC assay and a relatively close effect to that of EGCG and vitamin C in DPPH assay (Table 2).

According to the *in vivo* results, *C. fistula* was able to increase the survival rate in worms subjected to juglone induced oxidative stress by 68% (Fig. 2). Furthermore, ROS levels were significantly lower in worms treated by CF compared to the control group by 73%, showing a good scavenging effect of ROS *in vivo*. Both effects could be related to the phenolic constituents of the extract. These effects were concentration-dependent, indicating good absorption and bioavailability of the extracts (Fig. 3), similar effects were also observed in *C. elegans* using a hydroalcoholic extract from *Schotia brachypetala* leaves and a hydroalcoholic extract from *Euterpe precatoria* fruits (Peixoto et al., 2016a; Sobeh et al., 2016).

In order to investigate the mechanism underlying the antioxidant effect of CF extract, DAF-16 transcription factor localization was assayed. DAF-16/FOXO transcription factor in *C. elegans* is responsible for the regulation of lifespan and several genes related to stress response and metabolism (Mukhopadhyay, Oh & Tissenbaum, 2006). DAF-16 pathway could be affected by germ-line signaling, caloric restriction, and sensory perception (Lin, Defossez & Guarente, 2000; Arantes-Oliveira et al., 2002; Alcedo & Kenyon, 2004). It could also be activated by a decrease in insulin/IGF-1 signaling or an increase in JNK signaling (Kimura et al., 1997; Oh et al., 2005). The up-regulation of SOD-3 expression in CF-treated worms by 67% (Fig. 5), and the nuclear translocation of DAF-16 confirm the involvement of DAF-16 pathway in the *in vivo* antioxidant activity. Similar results were also obtained from previous studies using plant extracts with potential antioxidant activities in *C. elegans* (Kampkötter et al., 2007b; Peixoto et al., 2016b; Wang & Wink, 2016).

The downregulation of HSP-16.2 expression induced by polyphenol rich plant extracts and isolated compounds have been correlated with enhanced stress resistance in *C. elegans* (Abbas & Wink, 2009; Abbas & Wink, 2010). In our study EGCG, used as a positive control, downregulated the expression of HSP-16.2 in stressed worms compared to the juglone treated control. *C. fistula* treatment was also able to downregulate the expression of HSP-16.2 at 100 µg/ml; the lowest concentration tested. However at 300 µg/ml, an upregulation of 57% was observed (Fig. 6), which could indicate a pro-oxidant activity of the extract. According to (Fonte et al., 2008), HSP-16.2 is able to partially suppress Aβ-induced paralysis through modulating Aβ oligomerization. The increased expression observed at 300 µg/ml might explain the results observed in the paralysis assay, where this treatment could markedly delay the paralysis of transgenic worms by ameliorating Aβ toxicity. The increased expression of HSP-16.2 chaperone protein could also be responsible for decreasing the formation of polyQ40 aggregates, observed in polyQ40 assay, by preventing misfolding and refolding of the formed protein and its subsequent aggregation (Vos et al., 2010).

In order to explore other mechanisms that might contribute to CF antioxidant effects, the GST-4 assay was performed. Glutamylcysteine synthetase (gcs-1) and gst-4 are regulated by SKN-1/NRF2 transcription factor, which is considered a main regulator protein for Phase II detoxification mechanism in *C. elegans*, working in parallel with DAF-16/FOXO pathway (Salgueiro et al., 2017). Results show a substantial decrease in GST-4 levels in CF groups, which was significantly close to the EGCG group, compared to the control group (Fig. 7). Downregulation of GST-4 is correlated with lower oxidative stress as reported by a previous study that tested the effect of *G. biloba* extract and found its ability to decrease the expression of GST-4 enzyme under both normal growth and oxidative stress conditions (Kampkotter et al., 2007a).

Since a correlation is known between oxidative stress and neurodegeneration (Bonfili et al., 2018), the effect of CF was also tested on neurodegeneration models through examining the formation of polyQ and Aβ aggregates in transgenic worms. PolyQ aggregate formation is linked to several neurodegenerative diseases like Huntington’s disease. This disease is related to abnormal increase and repetition of the trinucleotide (CAG) in the Huntingtin gene. This leads to the formation of a mutant protein, with a high number of glutamine residues. This protein forms aggregates with subsequent neuronal dysfunction (Weber et al., 2014) and severe functional loss in the central nervous system.

In the current study, CF was found effective in decreasing polyQ40 aggregate formation compared to the control group by 80%. This result matches the EGCG group, used as a positive control in the study (Fig. 8). Other studies also reported a decrease in polyQ40 aggregation by an anthocyanin-rich plant extract and an isolated polyphenol, resveratrol, present in grapes and red wine (Parker et al., 2005; Peixoto et al., 2016b).

Several polyphenols interfere with the polymerization of Aβ in *C. elegans* (Abbas & Wink, 2010). To further investigate the extract’s effect on counteracting Aβ toxicity using worms expressing human Aβ peptide, paralysis assay was done.

**Figure 9:**
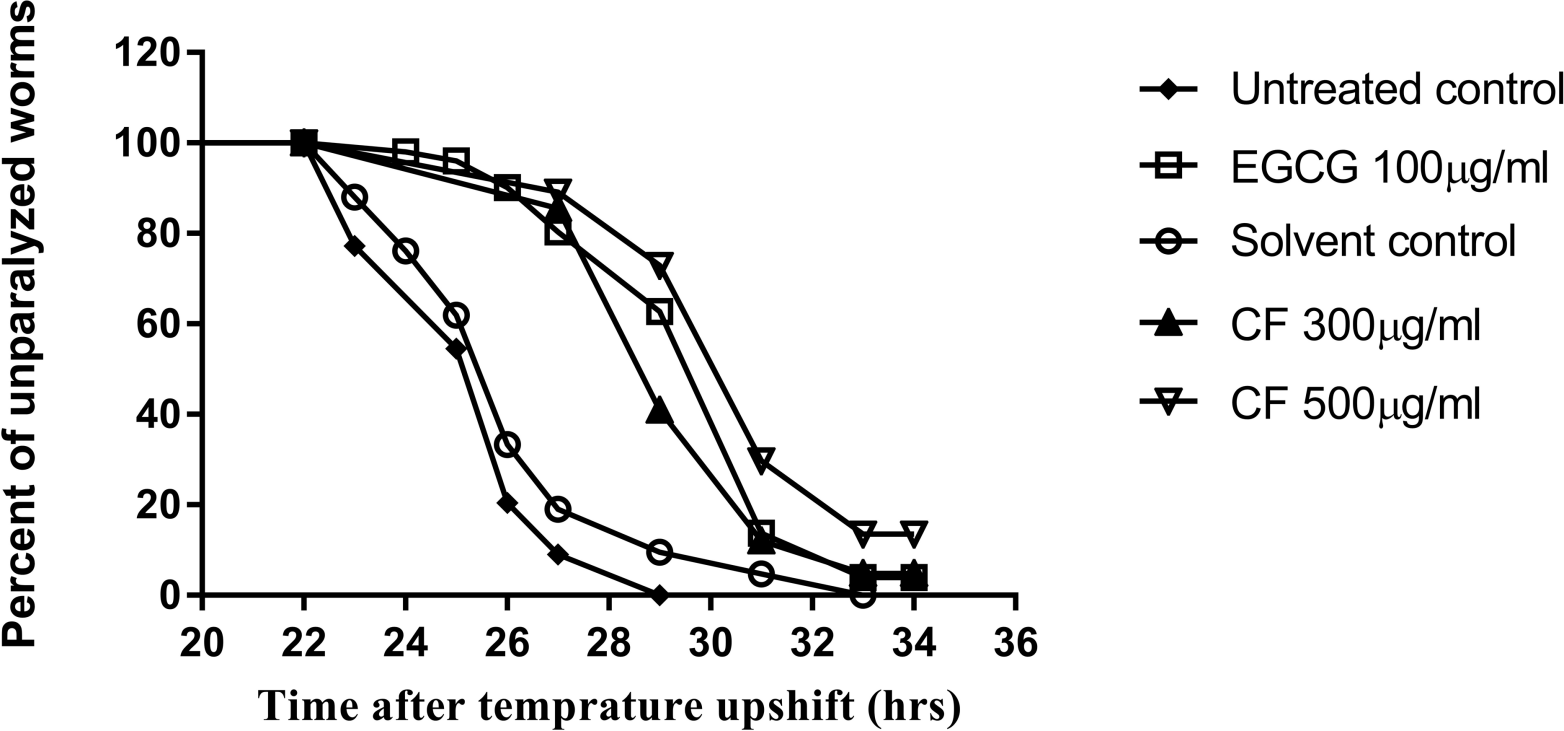
Effect of *C. fistula* extract on A*β* induced paralysis in *C. elegans*. Results of paralysis assay using CL4176 worms. CF 300 and 500 µg/ml treated worms showed slower paralysis compared to solvent control group.

CL4176 worms become paralyzed due to the expression of human Aβ_1−42_ peptides in their muscle cells, which also leads to further oxidative stress (Drake, Link & Butterfield, 2003). CF showed a concentration dependent delay in PT_50_ compared to the negative control group (Table 3). The result obtained for the highest concentration used of CF was 5 h, close to that of the EGCG group, 7 h (Table 3, Fig. 9). The anti-Alzheimer effect appears plausible as oxidative stress enhances the damage of human Aβ_1−42_ in worms, whereas treatment with the antioxidant vitamin E could prevent this effect (Behl et al., 1992; Yatin, Varadarajan & Butterfield, 2000). Furthermore, polyphenols can interact with many other proteins involved in metabolic and regulatory networks, because they can form several hydrogen and ionic bonds with proteins, thus modulating their 3D structures and bioactivities (Wink, 2015).

## Conclusions

In conclusion, the current study demonstrates the antioxidant and potential neuroprotective activities of the *Cassia fistula* extract in *C. elegans*. The extract was able to increase stress resistance and the expression of stress response genes, like SOD-3, in a DAF-16 dependent manner. In addition, the extract was also able to decrease the expression of HSP stress gene after exposure to oxidative stress. These results were confirmed by the ability of the extract to enhance nuclear localization of DAF-16 transcription factor. Possible involvement of SKN-1/NRF2 pathway was also demonstrated by a decrease in GST-4 levels. Neuroprotective effects of the extract were shown by a decrease in the formation of polyQ40 aggregates and a decrease in paralysis, probably due to lower Aβ aggregates formation. Further studies are required to study the exact molecular mechanism by which the extract exerts these effects, and to test for its efficacy and safety on higher model organisms.

##  Supplemental Information

10.7717/peerj.5159/supp-1 Data S1 Raw data

## Additional Information and Declarations

### Data Availability

The following information was supplied regarding data availability: The raw data is included as Data S1.
